# Variability in catheter-associated asymptomatic bacteriuria rates among individual nurses in intensive care units: An observational cross-sectional study

**DOI:** 10.1371/journal.pone.0218755

**Published:** 2019-07-10

**Authors:** Olga Yakusheva, Deena K. Costa, Kathleen L. Bobay, Jorge P. Parada, Marianne E. Weiss

**Affiliations:** 1 School of Nursing, School of Public Health, Institute for Health Policy and Innovation, University of Michigan, Ann Arbor, MI, United States of America; 2 School of Nursing, Institute for Health Policy and Innovation, University of Michigan, Ann Arbor, MI, United States of America; 3 School of Nursing, Loyola University Chicago, Chicago, IL, United States of America; 4 Department of Infection Control, Loyola University Medical Center, Chicago, IL, United States of America; 5 Marquette University College of Nursing, WI, United States of America; National Yang-Ming University, TAIWAN

## Abstract

Catheter-associated asymptomatic bacteriuria (CAABU) is frequent in intensive care units (ICUs) and contributes to the routine use of antibiotics and to antibiotic-resistant infections. While nurses are responsible for the implementation of CAABU-prevention guidelines, variability in how individual nurses contribute to CAABU-free rates in ICUs has not been previously explored. This study’s objective was to examine the variability in CAABU-free outcomes of individual ICU nurses. This observational cross-sectional study used shift-level nurse-patient data from the electronic health records from two ICUs in a tertiary medical center in the US between July 2015 and June 2016. We included all adult (18+) catheterized patients with no prior CAABU during the hospital encounter and nurses who provided their care. The CAABU-free outcome was defined as a 0/1 indicator identifying shifts where a previously CAABU-free patient remained CAABU-free (absence of a confirmed urine sample) 24–48 hours following end of shift. The analytical approach used Value-Added Modeling and a split-sample design to estimate and validate nurse-level CAABU-free rates while adjusting for patient characteristics, shift, and ICU type. The sample included 94 nurses, 2,150 patients with 256 confirmed CAABU cases, and 21,729 patient shifts. Patients were 55% male, average age was 60 years. CAABU-free rates of individual nurses varied between 94 and 100 per 100 shifts (Wald test: 227.88, P<0.001) and were robust in cross-validation analyses (correlation coefficient: 0.66, P<0.001). Learning and disseminating effective CAABU-avoidance strategies from top-performers throughout the nursing teams could improve quality of care in ICUs.

## Introduction

Catheter-associated urinary tract infections (CAUTI) are the most common hospital-acquired infection, contributing to increased mortality, morbidity, and costs in the United States and internationally [1, 2]. In intensive care units (ICUs), the rate of CAUTI is nearly twice as high as other device-associated infections (blood-stream infection and pneumonia), ranging as high as 4.9 CAUTIs per 1,000 catheter days in trauma and burn ICUs [3]. Not only is CAUTI the leading cause of secondary blood stream infection with an estimated 10% mortality risk in ICUs, but the associated routine antibiotic utilization for catheter associated asymptomatic bacteriuria (CAABU) contributes to increase in antibiotic resistant nosocomial infections [4].

Catheter-associated asymptomatic bacteriuria (CAABU) is the most important risk factor for CAUTI in ICUs. Urinary catheters are used routinely in ICUs for patients with limited mobility, especially if urine must be kept away from wounds (such as decubitus ulcers, surgical incisions, or burns), and for accurate monitoring of urinary output [5]. Fifteen percent of patients catheterized for more than 2 days will develop CAABU [6], and a patient’s risk of developing a symptomatic infection increases 3%-7% each day a urinary catheter remains [7]. The core components of CAUTI prevention bundles aim to reduce CAABU by using catheters only if indicated, and ensuring aseptic insertion, appropriate maintenance care, timely catheter removal, and post-removal care [8–11].

Nurses comprise the majority of the clinical workforce in ICUs and are directly responsible for catheter insertion, care, and surveillance. Recognizing the importance of nurses in infection prevention, the National Quality Forum, the leading non-profit organization focusing on healthcare quality in the US, includes CAUTI among nurse-sensitive infection measures [12]. While the decision to insert and remove a catheter is usually made by a physician, nurses provide the majority of hands-on catheter care, including insertion, maintenance, removal, and post-removal care. Many hospitals have also adopted nurse-driven catheter removal protocols that require nurses to communicate daily with physicians to confirm the medical necessity for the catheter and remove the catheter unless counter-indicated [13]. An evidence-based CAUTI prevention practice checklist for use in hospitals has been endorsed by the American Nurses Association [9]. Yet, breaks of evidence-based catheter care protocols can occur, putting patients at an increased risk of CAABU and CAUTI [11, 14, 15].

Existing studies linking reduced hospital-acquired infections to nursing care in ICUs have focused on nurse staffing and the work environment characteristics measured at the aggregated hospital- or unit-level [16–23]. However, individual nurses implement the prevention practices at an individual patient-level. We do not know whether there is nurse-level variability in CAABU-avoidance effectiveness. In non-ICU acute care settings, nurse-level variability in patient outcomes was recently reported for the outcomes of clinical condition improvement and patient-reported readiness for discharge [24, 25]. This area of research is important because, if clinician-level variability exists, some patients may inadvertently be receiving low quality care–even in high-performing well-staffed organizations or ICUs. Therefore, this study aimed to measure the variability of CAABU outcomes among individual ICU nurses.

## Materials and methods

### Design

This retrospective observational cross-sectional exploratory analysis of data from electronic health records (EHR) used a split-sample design for estimation and cross-validation of individual nurse CAABU-avoidance rates. We estimated nurse-level variability by applying an individual performance measurement method from the field of education economics, called Value Added Modeling (VAM) [26]. This method allowed for the calculation of risk-adjusted aggregated patient outcomes at the individual nurse level [24]. The study was approved by the University of Michigan Institutional Review Board (IRB) and the study site’s IRB (refer to Supporting File S1 Text). Under a limited data use agreement between the University of Michigan and the study site, data for the study were extracted by hospital information technology services, partially anonymized, curated, and provided to the research team via secure file transfer. The study team received partially anonymized data after all direct patient and nurse identifiers (names, social security numbers, addresses, etc.) were removed. Patients’ ICU encounter record numbers, nurses’ numeric EHR identification codes, ICU admission and discharge dates, and the dates and times of the CAABU/CAUTI events with the corresponding ICU encounter record numbers were preserved because these data were needed for temporally linking individual nurses with individual patients’ CAABU events. A waiver of patient consent was obtained for this secondary analysis of existing routinely collected electronic data. The study followed the STROBE reporting guidelines for observational studies (refer to S1 Table).

For each individual patient, data elements included: 1) a list of all ICU shifts where the patient had a catheter inserted or indwelling during the shift, with the date and an indicator of day/night shift, 2) nurse login identifiers by shift during ICU stay, 3) if any, a confirmed positive laboratory report of bacteriuria with the timestamp, 4) if any, a confirmed CAUTI report with the timestamp, and 5) patient clinical and demographic characteristics. There were no missing data.

### Setting

The study site was a 559 bed academic medical center in the Midwestern US with 8 ICUs and approximately 7,500 annual adult ICU encounters. Two adult ICUs, a medical ICU (MICU) and a neurological ICU (NeuroICU), with the most frequent use of indwelling catheters and highest CAABU rates were nominated by the site’s infection prevention team for inclusion in this study. During the data extraction period, the two study ICUs were similar (MICU: 19 beds, 1,204 annual discharges; NeuroICU: 16 beds, 1,164 annual discharges). The ICU length of stay (LOS) was higher in the MICU than in NeuroICU (5.5 vs 4.6 days). The hospital used 12-hour nursing shifts (7 a.m. to 7 p.m.) as their ICU staffing schedule and a 1 nurse to 2 patients ICU staffing ratio. The hospital had a nurse-driven decatheterization protocol [13] in place; nurses were trained in CAUTI-prevention evidence-based practices annually.

The hospital used standard United States Center for Disease Control and Prevention (CDC) criteria for CAABU/CAUTI identification [7]. For CAABU, the following must be present: 1) at the time of urine sample, patient had an indwelling urinary catheter in place for at least 48 hours and the catheter was still present on the day of the event or removed during prior 24 hours; and 2) positive urine culture with bacteria of at least 105 CFU/ml [27]. For CAUTI, the patient must also have: 3) at least one symptom (fever >38.0 C, suprapubic tenderness, costo-vertebral pain or tenderness, urinary urgency, frequency, dysuria). The hospital used infection surveillance software called MedminedTM. The software tracks all positive urine cultures in the hospital’s EHRs, applies CDC criteria 1 (catheterization) and 2 (bacteriuria threshold), and creates a report of CAABU cases [28]. As part of regulatory requirements for reporting hospital-acquired infections, the electronically reported CAABU cases were then manually reviewed for the presence of CDC criterion 3 (symptoms) by the hospital’s infection prevention team, and confirmed CAUTI cases were recorded.

### Sample

We included all inpatient admissions to the MICU and NeuroICU between July 1 2015 and June 31 2016 for patients who were 18 or older at the time of admission and had an indwelling or intermittent urinary catheter while in ICU. The study sample included 2,184 patients and 21,729 ICU patient-shift encounters, defined as a patient occupying a bed during a 12 hour shift. Each patient-shift encounter represented a data point for the analyses. Because the study focused on CAABU prevention activities in ICUs, we excluded 34 patients who had a report of positive CAABU or CAUTI prior to or at admission to the ICU. For patients who had a report of a positive CAABU or CAUTI at any point during the ICU stay, we retained only patient-shift encounters that occurred prior to the date and time of the report. The final analysis sample included 17,331 patient-shift encounters (2,150 patients). ICU patient-shift encounters with hospitalization discharge dates during the first six months of the data extraction timeframe from July 1 to December 31 of 2015 (8,572 patient-shift encounters, 1,087 hospitalizations) were used as the estimation dataset; the second six months of data from January 1 to June 31 2016 (8,759 patient-shift encounters, 1,063 hospitalizations) were used for cross-validating the estimates.

The nurse sample included 110 full-time registered nurses (57 nurses in MICU; 53 nurses in NeuroICU) who provided care during the ICU patient-shift encounters included in the final sample. Float nurses were excluded. We conducted post-hoc power analysis for the minimum number of patient-shift encounters per nurse required to detect an individual nurse effect on CAABU equal to 0.05705 (one-half the observed CAABU rate of 0.1141 in the estimation sample). Using a one-sample proportion test against the alternative of 0.000001 (approximating zero), 45 patient-shift encounters per nurse were required to achieve 0.80 power and 0.05 one-tailed significance in the estimation dataset. We excluded 16 nurses who were linked with fewer than 45 patient shifts during the first six months of the study since this presented too few encounters to appropriately classify their CAABU-free outcomes, thus yiedling the final nurse sample of 94 registered nurses (45 from Neuro ICU and 49 from MICU). Of the 94 nurses in the estimation set, 89 were also in the validation set (5.6% loss to follow-up).

### Measures

#### Outcome

For each patient-shift encounter, the CAABU-free outcome was defined as a 0/1 indicator variable equal to 1 if there was no record in the patient’s EHR of a confirmed CAABU-positive urine sample collected between 24–48 hours following the end of the encounter, and zero otherwise (if there was a record of a positive CAABU specimen). The event window of 24–48 hours after the end of shift is consistent with the CDC’s infection location attribution rule for multiple transfers that specifies a 24 hour minimum gap and a 48 hour maximum limit on infection attribution [7]. There were only 3 confirmed CAUTIs during the 12-month study period. This outcome was not included in the analyses.

#### Exposures

Per the VAM methodology described below, individual nurses were the exposures. Each nurse was assigned a unique anonymous indicator variable differentiating the nurse from the other nurses in the sample (a total of 94 indicators). For every patient-shift encounter, the nurse indicators of all nurses who provided care to the patient during the encounter were set to 1, the indicators of all other nurses (who did not take care of the patient during the shift) were set to 0.

### Statistical analyses

#### Descriptive statistics

Descriptive statistics for the sample were calculated as means and standard deviations for continuous variables and counts and percentages for categorical variables.

#### Estimation of nurse-level CAABU-free outcomes

The CAABU-free outcomes of individual nurses were estimated on the estimation dataset of 8,572 patient-shift encounters linked to 94 nurses in the 2015 sample.

The estimation model followed the VAM methodology [24–26]. The dependent variable was the encounter-level CAABU-free indicator. We used logistic regression to account for the dichotomous outcome variable. To model the nurses, 93 nurse indicator variables were entered in the model as separate covariates (one nurse indicator was randomly selected as the reference category). Modelled this way, each CAABU outcome was equally associated with all of the nurses who cared for the patient during the prior 24 to 48 hour period. This way, the approach accounted for the team-based nature of nursing care delivery.

Because the likelihood of developing CAABU can depend on a patient’s internal factors, we adjusted for patient clinical acuity and demographic factors, including: an indicator for the male gender [female gender was the reference category], a continuous variable for age (in years), twenty-one indicators for the patient’s Major Diagnostic Category (MDC) from primary discharge ICD-9/10 codes [MDC = “DDs of Nervous System” was the reference category],[29–31] an indicator for a surgical admission [medical admission was the reference category], and an indicator for previous hospitalization within 30 days prior to the current admission [no prior admission within 30 days was the reference category]. We did not have data on patient race or ethnicity, education, employment, or income; we included patient’s type of insurance (Private/Medicare/Medicaid/Uninsured [reference]) as a proxy for socioeconomic status and access to care.

To adjust for correlated effects in CAABU occurrence in space proximity and over time, we included shift-level control variables: a continuous variable for the number of hours the patient was in the ICU prior to the beginning of the shift, six indicators for the day of the week [Sunday was the reference category], and an indicator for a day shift [night shift was the reference category]. We also included an indicator for the MICU [NEURO was the reference category] to adjust the model for any differences in CAABU occurrence attributable to unit-level structure and care processes (e.g., staffing, work environment, hospital/unit infection prevention practices). The model was estimated with clustering at the patient-shift and the ICU- level (refer to S2 Table).

We then calculated the nurses’ CAABU-free rates. Using the fully adjusted VAM estimates of the regression coefficients of the nurse indicator variables, we computed CAABU-free rates as the predictive margins of responses for the nurse indicator variables [32, 33]. These predictive margins quantified the rate per 100 of each nurse’s patient-shift encounters where a CAABU-free patient remained CAABU-free 24–48 hours after the end of a shift. Because the estimation model included patient characteristics, shift-level controls, and ICU effects, the nurse-level CAABU-free rates were adjusted for these variables.

#### Measurement and testing of nurse-level CAABU-free rates

We examined the sampling frequency distribution of the nurse-level CAABU-free rates and reported their mean, standard deviation, skewness, kurtosis, and the Kolmogorov-Smirnov normality test.

To test the role of nurse variability for the CAABU outcome, we conducted two tests. First, using Cohen’s Chi-squared joint test of significance [34], we tested the null hypothesis that the VAM regression coefficients of the nurse indicator variables did not explain variance in the log-likelihood of the CAABU-free outcome, against the alternative hypothesis that they did. Second, we tested the null hypothesis that adding the nurse fixed effects did not improve the Area Under (Receiver Operating Characteristic) Curve (AUC) above the restricted model that included only the patient, shift, and ICU variables, against the alternative hypothesis that it did. Rejecting both of the null hypotheses would indicate significant nurse-level variability and its contribution to the CAABU outcome.

#### Validation of nurse-level CAABU-free rates

Before applying the cross-validation approach, we tested for random, versus selective, assignment of nurses to patients. If higher-performing nurses (higher CAABU-free rates) were selectively assigned to lower acuity patients, the VAM estimation approach would over-estimate nurse-level outcome differences. Vice versa, if higher-performing nurses were selectively assigned to higher-acuity patients, the estimated distribution of nurse-level CAABU rates from the VAM approach would understate true nurse-level outcome variability. Either biases would reduce the validity of the VAM estimates. Therefore, we tested the null hypothesis that the nurses’ CAABU-free rates from 2015 were not associated with the characteristics of the nurses’ assigned patients in 2016 (random assignment), using a Chi-square test for the patient’s MDC and insurance type and t-tests for all other patient variables. Failure to reject the null hypothesis of random assignment would support validity of the VAM measurement approach.

We then examined the concordance of nurse-level CAABU-free rate estimates generated by the VAM approach in the two independent patient samples (2015 and 2016). To do so, we estimated a second set of adjusted individual nurse CAABU-free rates using the 8,579 patient-shift encounters linked to the 89 nurses in both the 2015 and 2016 patient samples (refer to S3 Table) and calculated a weighted correlation coefficient between the 2015 and 2016 CAABU-free rate estimates of the nurses, weighted by the nurses’ patient-shift encounter numbers across both samples (nurses with more encounters received higher weights than nurses with fewer encounters). Using a t-test, we tested the null hypothesis that the weighted correlation coefficient was equal to zero, against the alternative that it was not. Rejecting the null hypothesis would support cross-sample validity of the VAM measurement approach. We also conducted a cluster analysis to explore any natural grouping of nurses based on their 2015 and 2016 adjusted CAABU-free rates, using kmedians (Canberra distance) [35] and the quartiles of the 2015 distribution as the initial grouping variable in 10,000 iterations. Because the estimation and validation samples included patient hospitalizations drawn independently from two different time periods, rejection of the null hypothesis in favor of a positive significant correlation coefficient in nurse-level outcomes across the years, and existence of consistently high and low outcome clusters were interpreted as supporting individual nurse effects on the CAABU-free patient outcome.

As the final validation analysis, we explored nurse transitions from having low CAABU-free outcomes to having high outcomes, and vice versa, between 2015 and 2016. We estimated a transition probability matrix [36] and examined the probabilities of remaining in the same quartile of the distribution of the adjusted individual CAABU-free rates in both years (a sign of consistent nurse CAABU-prevention performance), versus moving to another quartile in 2016. We estimated the matrix using a nurse-level multinomial logistic regression of a nurse’s adjusted CAABU-free outcome quartile in 2016 (a four-level categorical dependent variable) on the nurse’s adjusted CAABU-free outcome quartile in 2015 (three predictor variables for 2^nd^, 3^rd^, and 4^th^ quartile; first quartile was the reference category). We then calculated predictive margins of responses for the 2015 quartiles on each of the 2016 quartile transitions (including same-quartile transitions) and their 95% confidence intervals. For each of the transitions, we tested the null hypothesis that the probability of transition was equal to 0.25, against the alternative that it was greater than 0.25, using a one-tailed Chi-square test at the 5% significance level. The premise of the test was that if the observed differences in the nurses’ 2015 CAABU-free outcomes occurred due to chance, the nurses would have a random 25% chance of transitioning from any of the 2015 CAABU-free quartiles to any other the 2016 quartiles, and a 25% chance of staying in the same outcome quartile in both years. On the other hand, a same-quartile transition probability greater than the 25% random chance was interpreted as persistent individual nurse effects on the CAABU-free patient outcome.

All statistical analyses were performed in Stata15.0 statistical software. The programming code for replicating the analyses is included as a Supporting Information File (refer to Supporting File S2 Text).

## Results

The patient sample, described in Table 1, was 55% male; average patient age was 60 years old. There were no significant differences in patient characteristics between the estimation and validation sets except for small differences in the distribution of MDCs. In both the estimation and validation sets, an average nurse worked about 100 ICU shifts over the six-month period and cared for 2.2 patients per shift, for the aggregate total of 220 patient-shifts per nurse in each set. There were 124 confirmed CAABU cases (11.41%) in the estimation sample, with a non-significant elevation to 137 CAABU cases (12.89%) in the validation sample.

**Table 1:** Sample characteristics.

	Estimation Set (Jul 1 –Dec 31, 2015)	Validation Set (Jan 1 –Jun 31, 2015)	P* Value
**Patients, N**	**1,087**	**1,063**	
Sex, n (%)			0.549
Male n (%)	604 (55.57)	577 (54.28)	
Female n (%)	483 (44.73)	486 (45.72)	
Age, Mean (SD)	59.78 (16.85)	60.44 (16.37)	
Type of insurance, n (%)			0.306
Private n (%)	375 (31.74)	327 (30.76)	
Medicaid n (%)	537 (49.40)	522 (49.11)	
Medicare n (%)	170 (15.64)	164 (15.43)	
Other n (%)	25 (2.30)	29 (2.73)	
Uninsured n (%)	10 (0.92)	21 (1.98)	
Length of hospitalization, Mean (SD)	8.93 (10.09)	9.11 (10.52)	0.676
Number of days in intensive care, Mean (SD)	6.73 (6.38)	6.91 (7.02)	0.521
Intensive care unit (ICU) type, n (%)			0.762
Medical	516 (47.47)	512 (48.17)	
Neurological	558 (51.33)	535 (50.33)	
Both	13 (1.20)	16 (1.51)	
Admission type, n (%)			0.234
Medical	511 (47.01)	527 (49.58)	
Surgical	576 (52.99)	536 (50.42)	
Major Diagnostic Category, n (%)			<0.001
Diseases and Disorders (DDs) of Nervous System	314 (34.39)	331 (33)	
DDs of Eye	1 (0.11)	0 (0.00)	
DDs of Ear, Nose, Mouth And Throat	20 (2.19)	27 (2.69)	
DDs of Respiratory System	102 (11.17)	104 (10.37)	
DDs of Circulatory System	86 (9.42)	79 (7.88)	
DDs of Digestive System	36 (3.94)	54 (5.38)	
DDs of Hepatobiliary System And Pancreas	36 (3.94)	24 (2.39)	
DDs of Musculoskeletal System And Connective Tissue	93 (10.19)	86 (8.57)	
DDs of Skin, Subcutaneous Tissue And Breast	2 (0.22)	3 (0.3)	
DDs of Endocrine, Nutritional And Metabolic System	24 (2.63)	34 (3.39)	
DDs of Kidney And Urinary Tract	16 (1.75)	14 (1.4)	
Pregnancy, Childbirth And Puerperium	3 (0.33)	5 (0.5)	
DDs of Blood and Blood Forming Organs and Immunological Disorders	10 (1.1)	11 (1.1)	
Myeloproliferative DDs (Poorly Differentiated Neoplasms)	27 (2.96)	31 (3.09)	
Infectious and Parasitic DDs (Systemic or unspecified sites)	97 (10.62)	154 (15.35)	
Mental DDs	1 (0.11)	2 (0.2)	
Alcohol/Drug Use or Induced Mental DDs	5 (0.55)	3 (0.3)	
Injuries, Poison And Toxic Effect of Drugs	27 (2.96)	25 (2.49)	
Factors Influencing Health Status and Other Contacts with Health Services	2 (0.22)	1 (0.1)	
Multiple Significant Trauma	3 (0.33)	5 (0.5)	
Human Immunodeficiency Virus Infection	8 (0.88)	10 (1)	
MDC Category Missing**	174 (16.01)	60 (5.64)	
Confirmed hospital-acquired CAABU, n (%)			0.293
No	963 (88.59)	926 (87.11)	
Yes	124 (11.41)	137 (12.89)	
**Nurses, N**	**94**	**89**	
ICU type, N(%)			0.854
Medical, n (%)	49 (52.13)	47 (52.81)	
Neurological, n (%)	45 (47.87)	42 (47.19)	
Number of shifts, Mean (SD)	102.59 (38.40)	103.29 (39.10)	0.924
Number of patients per shift, Mean (SD)	2.12 (.38)	2.12 (0.39)	0.847
Number of patient-shifts, Mean (SD)	217.26 (83.69)	221.16 (85.49)	0.758

*P-value of the difference between the estimation sample and the validation sample.

**MDC groupings are based on principal MS-DRGs (Medicare Severity-Diagnosis Related Groups) diagnosis. Per CMS rule, when an MS-DRG diagnosis is ungroupable (e.g., a rare disease) or when the patient was hospitalized for an operating room procedure unrelated to their diagnosis, the MS-DRG for that encounter is assigned a three digit code between 981–999, and the MDC category is coded as “Missing”.

**Fig 1:**
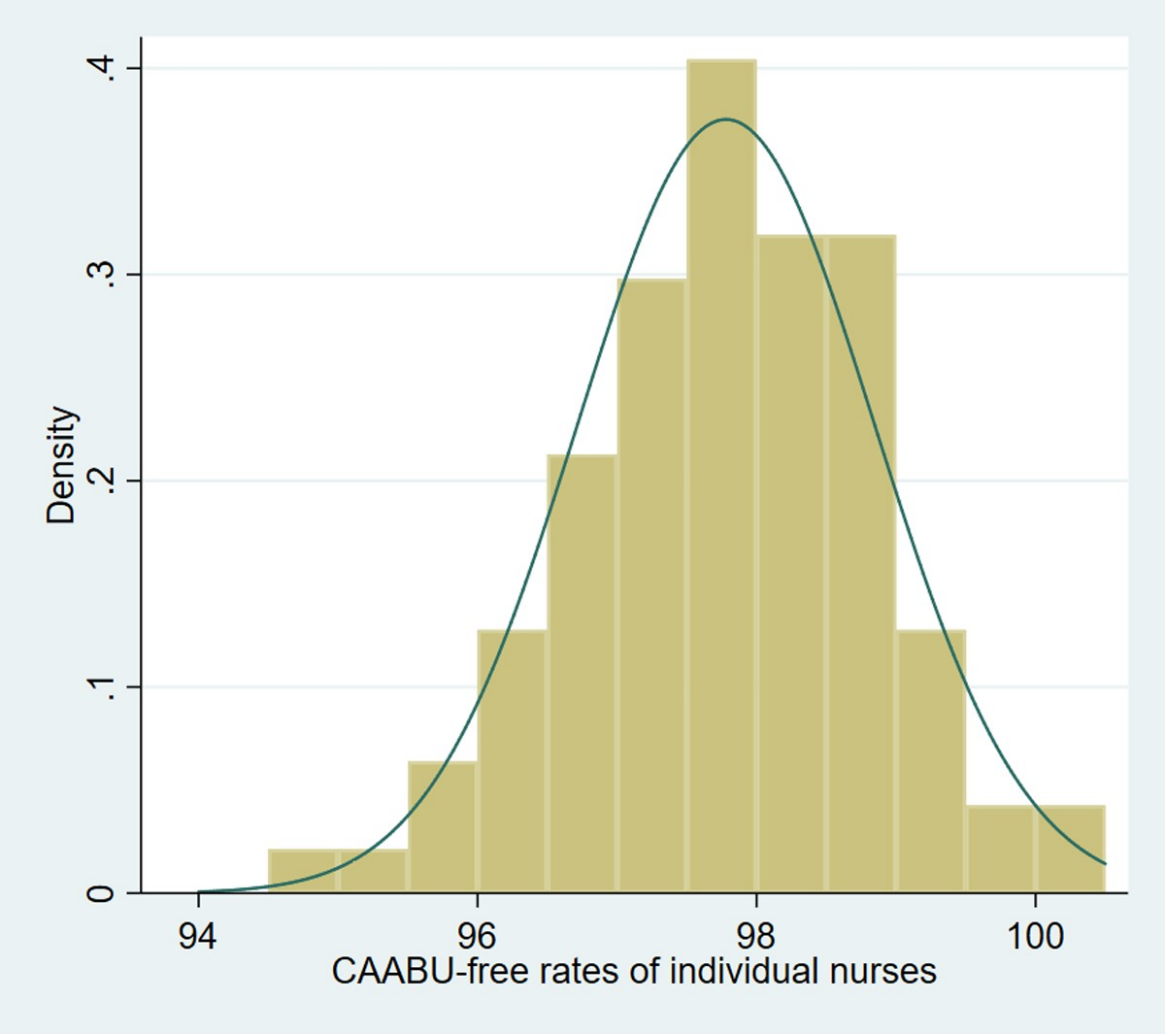
Distribution of adjusted individual CAABU-Free Rates (94 nurses in the estimation set, 2015). Catheter-associated asymptomatic bacteriuria (CAABU)-free rates per 100 intensive care patient shifts; Normal distribution superimposed.

In the estimation set, the frequency distribution of adjusted individual nurse CAABU-free rates had a sample average of 97.80, sample median of 98.00, and ranged from 94.20 to 100 per 100 patient-shifts. Among the 24 nurses in the lowest quartile (94.20 to 96.96) of the adjusted nurseCAABU-free rates, the average negative deviation from the sample median was -1.76 (SD = 0.67) CAABU-free cases per 100 shifts; among the 23 nurses in the highest quartile (98.77 to 100), the average positive deviation was 1.20 (SD = 0.45) CAABU-free cases per 100 patient-shifts. The cumulative density function (Fig 1) was approximately symmetrical (skew-test = -0.16) and had a slight positive kurtosis (3.23). The Kolmogorov-Smirnov test failed to rejected normality (P = 0.58). In hypotheses testing, the nurse fixed effects were jointly significant in predicting the CAABU outcome at p<0.001 (Chi-square test = 227.88), and nurse indicator variables increased the AUC statistics from 0.72 to 0.77 (P<0.001) supporting the contribution of nurse variability to CAABU-free outcomes.

**Fig 2:**
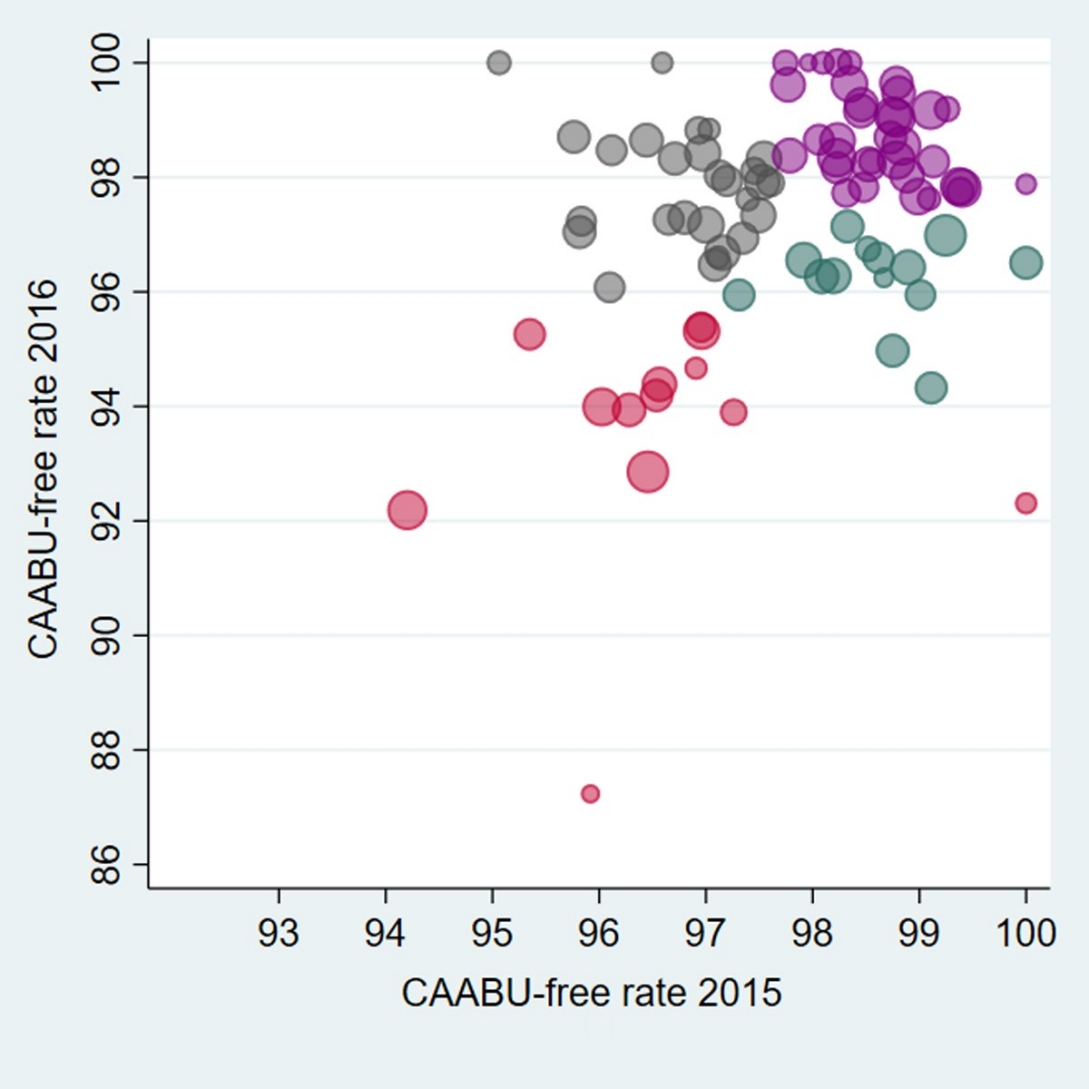
Cross-validation of individual CAABU-free Rates (89 nurses, estimation (2015) vs. validation (2016) set). Each point is a nurse; size reflects the number of patients per nurse; color indicates grouping by exploratory cluster analysis.

In cross-validation analyses using 89 nurses who cared for patients in both 2015 and 2016, 2015 nurse productivity was non-significantly associated with patient characteristics (age: t = -0.05, P = 0.96; sex: t = 0.23, P = 0.82; type of insurance: Chi-squared = 0.56, P = 0.69; surgical service: t = 1.85, P = 0.07; prior hospitalization: t = -0.16; P = 0.88; MDC: Chi-squared = 0.99, P = 0.47), failing to reject random patient-nurse assignment in this sample. CAABU-free rates obtained using the 2016 validation set were significantly and positively correlated with the rates obtained using the 2015 testing set (correlation coefficient 0.66, P<0.001). Exploratory cluster analysis (Fig 2) identified four non-overlapping clusters: 21 nurses had relatively high adjusted individual CAABU-free rates in both years (Cluster 1), 52 nurses with relatively high outcomes in one of the years (Clusters 2 & 3), and 16 nurses with relatively low adjusted CAABU-free rates in both years (Cluster 4).

Results of the transition probability analysis are displayed in Table 2. The probability of remaining in the lowest quartile was the only one greater than random chance (14 out of 24 low-preforming nurses in 2015 remained low-preforming in 2016, 0.57; 95% CI: 0.36–0.77); transition probabilities to and from the other quartiles were not significantly different from the 0.25 random chance.

**Table 2:** Probabilities and 95% confidence intervals, for the matrix of nurse transitions between CAABU-free quartiles from 2015 to 2016, n = 89 nurses.

		Nurses’ CAABU-free rates in the 2016 sample			
		87.23–96.47 Quartile 1	96.50–97.82 Quartile 2	97.84–98.65 Quartile 3	98.66–100 Quartile 4
**Nurses’ CAABU-free rates in the 2015 sample**	94.20–96.96 Quartile 1	0.568^#^ (.363 .771)	0.175 (.019 .332)	0.138 (-.003 .280)	0.119 (-.014 .252)
	96.97–97.99 Quartile 2	0.131 (-0.014 .277)	0.344 (.139 .547)	0.405 (.193 .616)	0.120 (-.019 .260)
	98.06–98.75 Quartile 3	0.180 (.017 .345)	0.204 (.032 .376)	0.266 (.077 .453)	0.350 (.147 .553)
	98.75–100 Quartile 4	0.142 (.004 .281)	0.279 (.101 .456)	0.285 (.106 .464)	0.294 (.113 .473)

Shown are the conditional probabilities of transitioning to each of the 2016 CAABU quartiles, conditional on starting from a given 2015 CAABU quartile; 95% confidence intervals are in parentheses. Calculations performed using predictive margins of responses from a nurse-level multinomial logistic regression of a nurse's 2016 quartile category on the nurse's 2015 quartile category, weighted by the number of patient-shift encounters of nurse.

# indicates that the probability of remaining in the lowest performing quartile was the only one significantly greater than random chance (25%) at .05 significance in a one-tailed Chi-square test.

## Discussion

### Main findings

We found evidence of significant variation in CAABU-free outcomes of individual nurses. While all nurses had very high CAABU-free rates (average CAABU-free rate of 98 per 100 catheter ICU patient shifts), the nurses in the top quartile had fewer than 1 new CAABUs per 100 shifts and the nurses in the bottom quartile had 3 to 5 new CAABUs per 100 patient-shifts. In cross-validation analysis using two independent patient samples, the association of individual nurse CAABU-free rates across independent patient samples was strong and a tendency toward persistently suboptimal outcomes in the lowest CAABU-free quartile was also evident.

### Interpretation of the findings

Although the observational nature of the study limits causal inference, the preponderance of evidence in this study supports individual nurse-level differences in achieving infection-free outcomes in ICUs. This finding is important because it can mean that some patients may have a lower or higher risk of developing bacteriuria, and possibly CAUTI, owing to individual nurses who provide their care. Few of the nurses had 100 percent CAABU-free outcomes and transition analysis revealed no consistent pattern of the same nurse being a superior performer in both years, suggesting a limit to how many adverse outcomes can be avoided even with most excellent nursing care. However, low infection-avoidance performance appeared to persist for some nurses over time, thus identifying a potential area for targeted individual intervention and improvement.

### Clinical implications and directions for future research

Scaling up the findings to the hospital level, our results indicate that bringing nurses in the lowest CAABU-free quartile up to the median might have helped avoid 93 of the observed 124 CAABU cases (-1.76 per 100 patient-shifts, times 220 patient shifts per nurse, times 24 nurses) for a 75 percent relative reduction during the six-month estimation sampling timeframe. Therefore, although a 100 percent infection prevention effectiveness is probably unachievable, reducing the lowest-end variability could have a significant positive impact on the overall quality of ICU care.

The observed variability in individual nurse CAABU-free outcomes might be interpreted through the lens of earlier evidence documenting variability in the use of evidence-based practice at the bedside. Nearly uniform (≥95%) infection prevention practice use by nurses is essential to the success of these efforts [11, 14, 37], yet individual nurse CAUTI-prevention care at the bedside is not always optimal. A 2011 survey of 1,653 ICUs in 965 US hospitals nation-wide showed that establishing hospital-wide infection prevention policies did not automatically ensure consistent clinician use of the prescribed infection-prevention practices at the bedside [11]. Although not all surveyed hospitals monitored individual clinicians, those that did reported low and variable practice use rates in ICUs, ranging from 6–27%,[11] echoing findings of an earlier nation-wide survey of hospital infection preventionists [38]. In one recent observational study [15], of 81 catheter insertions (65 patients) performed by emergency department (ED) nurses, researchers observed one (or more) major breaches of aseptic insertion technique in nearly 6 out of every 10 insertions, with many insertions involving multiple breaches.

To that extent, the results of our study highlight the need to recognize individual nurses as each being a unique and integral component of the care-delivery process. High individual CAABU-prevention effectiveness could be related to the consistent use of evidence-based practice, but it could also result from multiple other factors, including effective coping strategies to avoid burn-out and stress, teamwork and communication skills, and “work-arounds”, which make highly effective nurses thrive in the same environment where others might struggle. Being able to objectively measure and identify the entire distribution of nurse-level outcomes could inform periodic evaluation reviews while also providing the nursing team an opportunity to learn from nurses who have consistently high outcomes. Achieving high-quality patient care will require that high-performing nurses are recognized as role models with unique knowledge specific to the context of their unit, which they can share with the rest of the ICU nursing team.

The theoretical construct of individual clinician’s value-added contribution to patient outcomes has yet to be broadly applied to the interaction between nurses and patients. In the discipline of economics, VAM methods have found practical applications in educational assessment of teacher effectiveness [26]. With direct parallels to nurses and patient outcomes, VAM methods should be further adapted and developed to objectively demonstrate the importance of the nursing workforce to patient outcomes. The methodological advantage of VAM is the use of rigorous statistical techniques to separate the contribution of nurses from patient- and system-level factors, which is critical for healthcare delivery systems that want to maximize the value contribution of this important and costly human resource.

### Strengths and limitations

This study was a novel application of the Value-Added Modeling method from the field of economics to the measurement of risk-adjusted variability in infection-prevention outcomes of ICU nurses. Grounded in the fundamental economic construct of “Value-added”, or incremental increase in the value of output directly attributable to a productive input, we applied the method to the nurse-patient relationship by conceptualizing the contribution of a nurse’s care as value added to the quality patient outcome. This is a major innovation because it advances the study of healthcare workforce (nurses, physicians, etc.) beyond examining its aggregated measures like skill mix, education, or experience, to measuring the quality contributions of individual clinicians. Although bedside nursing care is provided by one nurse to one patient at a time, our study is the first to actually measure the value of the nursing infection-prevention practice at the individual nurse-patient level. An additional strength of the study is the use of time-stamped patient and nurse data which allowed for establishing direct linkages between individual patients and the specific nurses who provided their direct care throughout hospitalization, and to also correctly place each nurse’s care in relation to the time of occurrence of an adverse patient event. The use of the CDC’s infection attribution time window and access to verified CAABU data lend further strengths to our study methods and findings.

Nonetheless, there are several limitations that should be mentioned. First, unmeasured confounding is an issue in all observational studies. In our prior work in acute care settings, we found that higher-productivity nurses were assigned to higher-acuity patients [24, 25, 39, 40]. Although there was no evidence of non-random nurse-patient assignments in this study and we adjusted for many patient and shift-level potential confounders, unmeasured confounding may still be present, potentially weakening statistical signals from individual nurses. Despite unmeasured confounding, we were able to detect statistically significant differences in nurse-level outcomes, suggesting that there was something about specific nurses that contributed to more or less effective CAABU prevention. We cannot discern what, specifically, it was about each nurse that generated the nurse-level statistical signals observed in our analysis. It could be nurse experience, skill level, or knowledge of evidence-based practice. It could also occur due to factors outside of a nurse’s direct control, such as frequently working with a less experienced nurse assistant or providing care in a room with a poorly placed hand sanitizer. Yet, our approach is innovative and important, precisely because it allows us to begin to explore these currently unobserved and unknown factors that can occur at the individual nurse level and contribute to better, or worse, patient outcomes.

Second, it is extremely difficult, if not impossible, to discern which nurse or nurses contributed the most to the prevention of bacteriuria in a particular patient, because nursing care is 24 hours 7 days a week and fluid. Therefore, the approach of attributing a CAABU-free outcome to all of the nurses who cared for the patient during the prior 24–48 hours is consistent with how nursing care is delivered. At the same time, the equal distribution of credit for a CAABU outcome likely means that we report variability in individual nurse CAABU-prevention effectiveness conservatively.

The small number of confirmed CAUTIs in this sample precluded us from examining the association between individual nurses and CAUTI outcomes. Most likely owning to a routine treatment of CAABU with antibiotics, only 3 out of the 261 confirmed CAABU cases in our sample progressed to a CAUTI diagnosis. While being effective in avoiding a symptomatic infection, antibiotics also produce side effects and drug interactions, and their use contributes to development of antibiotic-resistant infections in hospitals [6]. Improving individual nurse CAABU-avoidance effectiveness has a potential to prevent CAUTIs, while also reducing the need for routine use of antibiotics.

There was variability among nurses in the number of patient shifts used in calculating individual CAABU-free estimates; however we eliminated nurses with small numbers of patients and weighted analyses to avoid undue influence from small numbers. We were limited in the number of patient outcomes (too few urinary tract infections; data on prescription antibiotics, costs, or post-discharge outcomes were not available). Due to de-identification, we were unable to identify repeat hospitalizations of the same patient. As with any single-facility study, the results may not be generalizable to other settings and patient populations.

### Conclusion

The objective of this study was to examine the variability in CAABU-free rates among individual ICU nurses. The finding that different nurses had different CAABU-free rates is a first step toward a better understanding of ICU care quality variability at the individual care provider-level and its potential role for patient outcomes. Learning and disseminating effective CAABU-avoidance strategies from top-performers throughout the nursing teams, and uncovering barriers to high infection-prevention performance, could improve quality of care in ICUs.

Our analytic approach can be adapted to other hospital-acquired infections (e.g. catheter-associated blood-stream infections, ventilator-associated pneumonia) and other nursing-sensitive patient outcomes (e.g., falls, pressure injuries) in both ICU and non-ICU settings. Further research in this area can inform new individual provider-level approaches that would capitalize on the knowledge of clinician-level variability to improve knowledge- and skill-sharing among peers and to inform strength-based clinical care teams. Added to the existing system-level efforts to improve staffing, work environment, and training, lessons learned from clinician-level variability in outcomes can help organizations ensure that high-quality health care is provided to each patient, every time.

## Supporting information

S1 Table STROBE statement: Checklist of items that should be included in reports of cross-sectional studies. (DOCX)

S2 Table Value added model, regression output, estimation set, n = 8,572 patient shift encounters. The table presents the regression betas (coefficients of the log-likelihood model) obtained from the estimation set, 2015. Estimates are obtained using a logistic regression with clustering at the patient-shift encounter level. [ref] = category excluded from model; [omitted] = category dropped due to collinearity and combined with the reference category during estimation.
(XLSX)

S3 Table Value added model, regression output, validation set, n = 8,759 patient shift encounters. The table presents the regression betas (coefficients of the log-likelihood model) obtained from the validation set, 2016. Estimates are obtained using a logistic regression with clustering at the patient-shift encounter level. [ref] = category excluded from model; [omitted] = category dropped due to collinearity and combined with the reference category during estimation.
(XLSX)

S1 Text Data request document for retrospective chart and/or material review studies. Institutional Review Board for the Protection of Human Subjects. Loyola University Chicago Health Sciences Division.
(DOC)

S2 Text Annotated Stata15.0 programming code for replicating the study results. (TXT)
