# Supplementary material for: Variability in catheter-associated asymptomatic bacteriuria rates among individual nurses in intensive care units: An observational cross-sectional study
Source: PLoS One. 2019 Jul 10;14(7):e0218755. doi: 10.1371/journal.pone.0218755 (PMC6619985; doi:10.1371/journal.pone.0218755)
Supplement: S1 Text — Institutional Review Board for the Protection of Human Subjects. Loyola University Chicago Health Sciences Division. (DOC) [file pone.0218755.s004.doc]

**Loyola University Chicago**

**Health Sciences Division**

**Institutional Review Board for the Protection of Human Subjects**

**Retrospective Chart and/or Material Review Studies**

The following information and template are to be used when submitting a request to conduct retrospective chart and/or material review studies.

1. Retrospective chart/existing materials reviews require:
   1. a written protocol (use the attached template),
   2. an IRB application through the information portal,
   3. IRB review and approval,
   4. waivers of consent and authorization,
   5. an approval letter provided to the keeper of the records.
2. Conditions of approval will be:
   1. You are bound by the usual and customary medical, legal and ethical considerations governing the confidentiality of the medical record.
   2. The data you collect may not be sold or given to any third party outside the scope of this submission unless it is to a journal for publication. Data to a journal for publication must be de-identified.
   3. The chart reviewed is to be identified on the data collection form by a unique code number and the master list kept under lock and key. If you are not collecting data elements that can directly or indirectly identify the chart reviewed then this requirement is not relevant.
3. The link of the patient to the project is to be destroyed when it is no longer necessary.

To waive consent, the IRB must find:

1. The study is of minimal risk and qualifies for expedited review 45CFR46.110, b-1, HHS Secretary Category).
2. The requirement for consent is waived if:
3. the research could not practicably be done without the waiver;
4. the waiver does not adversely affect the rights of the individual;
5. the research involves no more than minimal risk;
6. there is no information that would need to be provided to the individual.

(45CFR46.116, d1-4)

To waive authorization, the IRB must find:

1. The use or disclosure of protected health information involves no more than minimal risk to the individuals;
2. The alteration or waiver will not adversely affect the privacy rights and the welfare of the individuals;
3. The research could not practicably be conducted without the alteration or waiver;
4. The research could not practicably be conducted without access to and use of the protected health information;
5. The privacy risks to individuals whose protected health information is to be used or disclosed are reasonable in relation to 1) the anticipated benefits (if any) to the individuals, and 2) the importance of the knowledge that may reasonably be expected to result from the research.
6. There is an adequate plan to protect the identifiers from improper use and disclosure;
7. There is an adequate plan to destroy the identifiers at the earliest opportunity consistent with conduct of the research, unless there is a health or research justification for retaining the identifiers, or such retention is otherwise required by law;
8. There are adequate written assurances that the protected health information will not be reused or disclosed to any other person or entity, except as required by law, for authorized oversight of the research project, or for other research for which the use or disclosure of protected health information would be permitted by this subpart.

**LOYOLA UNIVERSITY CHICAGO**

**HEALTH SCIENCES DIVISION**

**MAYWOOD, ILLINOIS**

**Project Title**: Examining nurse-level differences in CAUTI rates in ICUs

**Principal Investigator**: Jorge Parada, MD, MPH, Professor, Stritch School of Medicine, (708) 216-3135, [jparada@lumc.edu](mailto:jparada@lumc.edu)

**Co-Investigator**:

Olga Yakusheva, PhD, Associate Professor, University of Michigan Schools of Nursing and Public Health, (734) 936-1327, (734) 647-2415, [yakush@med.umich.edu](mailto:yakush@med.umich.edu)

Marianne Weiss, DNSc, RN, Professor, Marquette University College of Nursing, (414) 288-3855, [Marianne.weiss@marquette.edu](mailto:Marianne.weiss@marquette.edu)

Kathleen Bobay, PhD, RN, Professor, Marcella Niehoff School of Nursing, (708) 216-5737, [kbobay@luc.edu](mailto:kbobay@luc.edu)

**1. Purpose / Rationale (appropriately referenced**): The purpose of this exploratory study is to obtain effect size estimates for the impact of individual nurse performance on occurrence of a hospital acquired infection, specifically catheter associated urinary tract infection (CAUTI) in ICU patients.

Healthcare-associated infections (HAIs) are some of the most frequent adverse event in healthcare, affecting about one in 20 hospital patients and costing the US healthcare system conservatively $10 billion a year.^1,2^ Central line-associated bloodstream infections (CLABSI), catheter-associated urinary tract infections (CAUTI) and ventilator-associated pneumonia (VAP) represent three of the most frequently occurring HAIs. These HAIs result in the highest number of deaths in intensive care units (ICUs),^3^ with CLABSI and VAP also being the most expensive at $40,000-45,000 per case.^2^ An estimated 65%-70% of CLABSIs and CAUTIs, and 55% of VAP cases, are reasonably preventable using current evidence-based prevention programs.^4^ Given that bedside nurses are responsible for monitoring and surveillance activities, this gives bedside nurses the ability to directly impact infection prevention leading to positive, or negative, patient outcomes.^5-7^

Studies linking reduced prevalence of HAIs to nursing care in ICUs have focused on nurse staffing and the work environment measured at the hospital- or unit-level.^8-17^ What remains unknown is whether, among nurses in the same ICU, some perform consistently better than others in terms of preventing HAIs; and, if such individual nurse-level performance differences in HAI-prevention exist, what factors contribute to high and low individual performance. Closing this gap could fundamentally change the way we think about HAI prevention, from the current one-size-fits-all recommendations to increase staffing and improve the work environment, to novel individual nurse-tailored approaches to ICU quality improvement to prevent HAIs.

In earlier peer-reviewed work, members of this team developed a novel approach of using electronic medical records to link nurses to the outcomes of patients they cared for. We showed that outcomes-based performance varied among nurses on the same unit, with patients receiving care from high-performing nurses experiencing shorter lengths or stay, lower costs, and reduced odds of 30-day readmission. However, the role of individual nurse performance has not been examined in critically-ill ICU patients who are at the highest risk for acquiring HAIs.

**2. Objective**: In this study, we will estimate the relationship of individual ICU nurse performance to a single HAI, specifically CAUTI in 2 ICUs.

The specific aim is t***o measure individual nurse performance on HAI prevention in ICUs.*** We will compute nurse-level *CAUTI performance scores*, adapting the risk-adjustment methodology used by Medicare’s Hospital Acquired Condition Reduction Program to the level of an individual nurse. The CAUTI performance score is an outcome-based performance score computed by linking individual nurses to all the patients for whom they provide care and using CAUTI occurrence as the patient outcome. Nurses with CAUTI scores in the top and in the bottom quartile will be categorized as “high” and “low” performers, with the middle quartiles categorized as “average performers.” We will not reveal the categorization to the nurses, managers, or administration.

**3. Methods and Material**: [Indicate: a) what will be reviewed, b) the criteria for pulling the charts for review, and c) how you will know what charts to review. Examples: ICD-9 diagnostic codes, departmental database, etc.]

Sample includes all patients admitted to the MICU and NeuroICU, who had an indwelling or intermittent urinary catheter while in ICU, over a 12 month period (July 1 2015 to June 30, 2016). The sample will also include all nurses providing care to these patients while in the ICU. Patients are selected by unit placement within the study time period, not by diagnosis.

We are requesting a linked patient- nurse file. For each patient admitted to the ICUs, we are requesting the EMR login ID number of each nurse providing care to the patient while in the 2 ICUs with the login timestamp and the occurrence of a CAUTI with its timestamp. CAUTI timestamps will be obtained through the entire hospitalization.

**4. Data Collected**

**For each individual patient, from the patient electronic record**:

**Main analysis variables**

a. Series of nurse login IDs with timestamps for all logins while a patient is in the ICU

b. lab report of UTI with timestamp

c. EMR fields indicating CAUTI (to be determined by IT staff)

**Control variables:**

**a**. ICU unit with entry and exit timestamp

b. Length of stay

c. Admission diagnosis

d. first 5 ICD codes

e. discharge diagnosis

f. demographic characteristics:(age in years, gender),

g. insurance type (private/Medicare/Medicaid/none),

h. an indicator for a surgical admission

1, an indicator for a hospitalization within 30 days prior to the index admission.

**5. Plan to Protect Patient Identifiers from Improper Use and Disclosure**:

LUMC IT staff will de-identify the nurse login ID before providing the dataset to the research team. Because we need timestamp information, these patient level data cannot be de-identified. The timestamp is a date/time field that is one of the HIPAA-defined identifiers. The time stamp is needed to determine the association of a nurse login to the development of a CAUTI (timestamp on lab report of CAUTI and nurse login will be used). A limited data use agreement will therefore be executed.

We are requesting waiver of consent:

1. The study is of minimal risk and qualifies for expedited review 45CFR46.110, b-1, HHS Secretary Category).

This study is a retrospective chart review.

2. The requirement for consent is waived if:

1. the research could not practicably be done without the waiver;

The collection of data is retrospective, therefore we are not able to contact patients retrospectively. In order to conduct the analysis effectively, we need all eligible patients.

1. the waiver does not adversely affect the rights of the individual;

We will not be collecting direct identifiers that would allow us to know who the patient is; we are collecting time-stamped information which could indirectly identify the patients. The information collected related to the patient is not-sensitive and could not be used to adversely impact the patient. Nurse identities are being protected through de-identification of their login number. Nurses will not therefore be informed of their nurse performance rating, an outcome of the study. Unit managers will not be informed about the results of the study or individual nurse performance rating.

1. the research involves no more than minimal risk;

There are no more than minimal risks – this is not an intervention study.

1. there is no information that would need to be provided to the individual.

(45CFR46.116, d1-4).

There is no information that would need to be provided to patients. The CAUTIs we are studying have already been identified and treated.

We are requesting waiver of authorization:

1. The use or disclosure of protected health information involves no more than minimal risk to the individuals;

This study is minimal risk. This is a retrospective study. Publications will not report any identifying information about the nurses or patients.

1. The alteration or waiver will not adversely affect the privacy rights and the welfare of the individuals;

There will be no disclosure of personally identifying information in publications or presentations. Data files will be held in secure computer files.

1. The research could not practicably be conducted without the alteration or waiver;

This is a retrospective study; it would be impossible to retrospectively obtain authorization.

1. The research could not practicably be conducted without access to and use of the protected health information;

In order to link individual nurse performance to patient level outcomes, we need to be able to have access to the patient level data on CAUTI and related descriptive data about the patient’s characteristics and hospitalization. This information is located in EMR files and nurse data is in the human resource files. Access to these data with appropriate protections is needed to conduct the study.

1. The privacy risks to individuals whose protected health information is to be used or disclosed are reasonable in relation to 1) the anticipated benefits (if any) to the individuals, and 2) the importance of the knowledge that may reasonably be expected to result from the research.
   1. There is an adequate plan to protect the identifiers from improper use and disclosure; Nurse login numbers will be de-identified by IT staff before providing to the research team. Patient direct identifiers will not be included in the dataset but time-stamped events will be included (admission, discharge date, diagnosis, CAUTI occurrences). We will therefore execute a limited data use agreement to cover protection and use of these data. Aggregate data will be reported in presentations and publications.
2. There is an adequate plan to destroy the identifiers at the earliest opportunity consistent with conduct of the research, unless there is a health or research justification for retaining the identifiers, or such retention is otherwise required by law;
   1. We will retain the data file for use in a larger analysis once grant funding is obtained. If funding is not obtained, these data file will be retained for 3 years or until publication of findings, and will then be deleted. If grant funding is obtained the limited data file will be incorporated into the full study file and retained through completion and publication from the full study.
   2. There are adequate written assurances that the protected health information will not be reused or disclosed to any other person or entity, except as required by law, for authorized oversight of the research project, or for other research for which the use or disclosure of protected health information would be permitted by this subpart.
   3. The data for this study will be used for the purposes indicated and will not be shared outside the research team.

**6. State When Patient Identifiers Will Be Destroyed**: We will retain the data file for use in a larger analysis once grant funding is obtained. If funding is not obtained, the data file will be retained for 3 years or until publication of findings, and will then be deleted. If grant funding is obtained the pilot data file will be incorporated into the full study file and retained through completion and publication from the full study.

**7. Describe Who Will Have Access to the Protected Health Information**: Dr Olga Yakusheva will be the primary analyst and the only researcher with direct access to the identified data file.

**8. Describe Use of the Data:** (publication, presentation, etc.) Publication, preliminary data for grant submission.

**9. Bibliography**:

References
